# Influence of Distraction Factors on Performance in Laparoscopic Surgery in Immersive Virtual Reality: Study Protocol of a Cross-Over Trial in Medical Students and Residents—DisLapVR

**DOI:** 10.2196/59014

**Published:** 2024-11-05

**Authors:** Laura Hanke, Richard Schulte, Christian Boedecker, Florentine Huettl, Patrick Saalfeld, Vuthea Chheang, Marlene Wessels, Christoph von Castell, Heiko Hecht, Christian Hansen, Hauke Lang, Tobias Huber

**Affiliations:** 1 Department of General, Visceral and Transplant Surgery University Medical Center of the Johannes Gutenberg-University Mainz Mainz Germany; 2 Institute of Simulation and Graphics Faculty of Informatics Otto-von-Guericke University Magdeburg Magdeburg Germany; 3 Department of Experimental Psychology Johannes Gutenberg-University Mainz Mainz Germany

**Keywords:** immersive virtual reality, distractions in surgery, laparoscopy, medical training, medical students, surgical education, surgical training, VR, cognitive load, multitasking, stress resilience

## Abstract

**Background:**

Working in an operating room (OR) is physically and mentally challenging: the operation itself demands the surgeon's full attention, while time and cost efficiency constraints, daily planning, and emergency care interfere with the procedure. Thus, multitasking becomes an integral surgical competence. This study aims to examine the effect of disruptions during surgery in a highly immersive virtual reality (IVR) operation environment combined with a virtual reality (VR) laparoscopy simulator.

**Objective:**

This study aims to identify distractions in the OR and their importance in the clinical setting.

**Methods:**

An IVR environment was created using a high-resolution, stereoscopic 360° video of the OR. Different distractions were identified, classified as auditory, visual, or audio-visual, and recorded accordingly. The surrounding was combined with a VR laparoscopic simulator. Participants—medical students and surgical residents—received proficiency-based training in basic laparoscopic skills and were blinded to the aim of the experiment. Following a cross-over design, each participant received a unique order of virtual distraction factors while performing tasks on the laparoscopic simulator. During the experiment, subjective passing of time, stress, heart rate, and visually induced motion sickness are recorded. After the experiment, validated questionnaires for usability, immersion, and stress were completed, as well as subjective evaluation of the distractions. The questionnaires used included the system usability scale, Self-Assessment Manikin score, National Aeronautics and Space Administration Task Load Index, and the immersion rating scale as described by Nichols. Performance in the laparoscopic tasks in relation to distractions will be evaluated by the Wilcoxon test and ANOVA for continuous variables. Subgroup analyses in regard to age, gender, and expertise (medical students vs surgical residents) are planned.

**Results:**

The described trial started in August 2022 and is ongoing. By July 2024, a total of 30 medical students and 9 surgeons have completed the study.

**Conclusions:**

We present a study protocol aiming to identify the impact of different disruptions in OR during laparoscopic training in IVR. Hence, it may lead to an improved awareness of distractions and facilitate accommodations toward an improved work environment. Prior research leads to the hypothesis that the performance of a more experienced surgeon is less impacted by distractions than the performance of inexperienced surgeons and medical students. Furthermore, we investigate which type of distraction has the largest impact on performance. With this knowledge, specific multitasking training can be devised, which may be particularly useful in medical education, for which VR might play a leading role. Additionally, workplace surroundings in the OR can be optimized with this knowledge.

**Trial Registration:**

German Registry for Clinical Trials DRKS00030033; https://drks.de/search/en/trial/DRKS00030033

**International Registered Report Identifier (IRRID):**

DERR1-10.2196/59014

## Introduction

Laparoscopic simulation has been shown to be beneficial in the training of surgical residents [1-3] and has been a mandatory part of surgical education in different countries. Following this example, an obligatory laparoscopic simulation curriculum consisting of 6 tasks was implemented in the Department of General, Visceral and Transplant Surgery, University Medical Center Mainz, Germany.

Surgery requires different professions to cooperate closely and efficiently to facilitate a smooth workflow while maintaining high levels of concentration on the procedure itself. Patient safety and cost efficiency demand swift procedures, which are regularly impaired or interrupted by various factors: emergency care, organizational tasks, and management of complications cannot be postponed in many cases; thus, multitasking becomes a surgical key competence. Moreover, teaching residents and medical students remains an integral part of working in an operating room (OR). In combination, these circumstances turn the OR into a bustling workplace with high physical and mental demands, rendering the ability to multitask a key surgical competence. Prior studies have identified and quantified distractions in the OR and examined their influence on surgeons [4-6]. Experienced surgeons were found to be more adept at handling distractions and were less impaired by them [7], suggesting this to be an ability that can be trained.

Laparoscopic simulation training usually takes place away from the hustle of everyday clinical work, yet structured training for multitasking or stress resilience has not been developed so far.

To test laparoscopic simulation in more realistic conditions, the institute designed a highly immersive virtual reality (IVR) application using high-resolution, stereoscopic 360° videos of the OR and displaying them on a head-mounted display (HMD; Vive Pro, HTC Corp) [8,9]. This application includes the surgery-unrelated background work in the OR including typical background noises and movements. In a prior study of our group, an IVR setting using 360° videos was deemed more realistic and was preferred by participants over an artificial virtual reality (VR) setting [10]. Furthermore, it has been previously shown that 360° video-based VR experiences enhanced memory recall, compared to conventional 2D videos [11], suggesting facilitated learning.

Prior studies could show that training in immersive laparoscopy induces a higher cognitive load, making this method already more demanding than conventional laparoscopy training [12]. Sankaranarayanan et al [13] have presented a simulated IVR program with similar goals and were able to show a significant difference in performance with or without distraction. As mentioned, our group was able to show in the past that IVR using real video material is preferred by users over simulated environments [10,14]. We aimed to create a realistic environment with realistic distractions, that is, phone calls or presentations of the next case, in contrast, other studies in the past have used unrelated distractions like music or other noises [13]. To achieve a realistic environment, different distractions were identified and classified as auditory (ie, a phone call), visual (ie, somebody walking through the field of vision), and audio-visual (ie, somebody presenting an emergency case including a computed tomography scan). These distractions were recorded and can be inserted into the simulation at any time. Participants can immerse themselves into this realistic environment and perform tasks on a laparoscopic simulator including haptic feedback (LapSim, Surgical Science, software version LapSim 2020.2). The simulator detects numerous parameters such as task completion time, tissue handling, amount of bimanual work, and instrument path length to calculate a performance score. Laparoscopy tasks are designed to train numerous abilities and competencies.

We present a protocol for a study aiming to identify the impact of different distractions during laparoscopic simulation in IVR. This study aims to identify distractions in the OR and their importance in the clinical setting.

## Methods

### Aims

**Figure 1:**
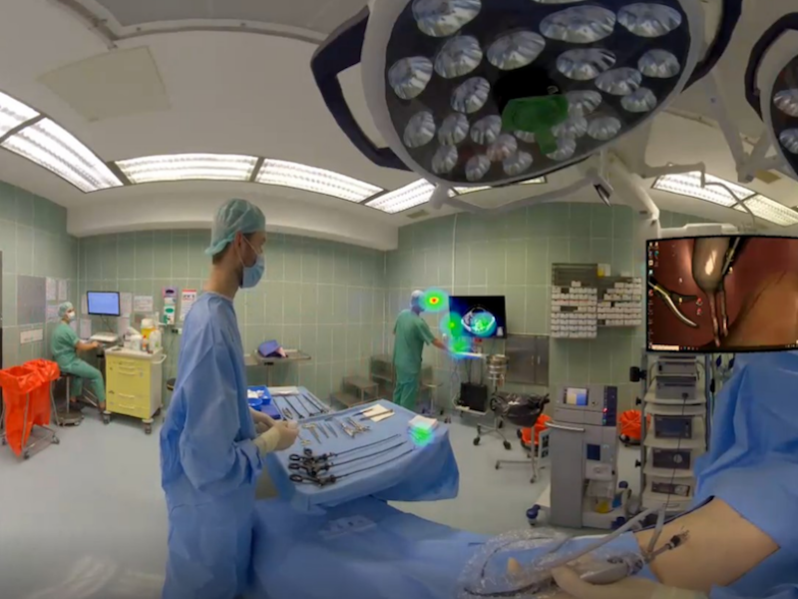
Part of the participant’s view in immersive virtual reality is shown. The scrub nurse is standing beside the participant. On the left edge of the picture, the laparoscopy monitor with an exercise is shown. In the background, a colleague is demonstrating a CT scan of the next emergency patient with a bowel obstruction (audio-visual distraction). CT: computed tomography.

This study aims to examine the influence of distractions on laparoscopic surgery in IVR. Therefore, a unique presentation sequence was created for each participant repeating the 6 tasks combined with 5 different distractions and without distractions. Each task will be performed with each distraction once and without distraction twice following a cross-over design. Figure 1 depicts one of the distractions in VR.

After completing the proficiency-based curriculum and passing the final tests, all surgical residents as well as residents in gynecology are eligible to participate in the study. As a second group, medical students, who complete the same curriculum with lower thresholds, are eligible to participate in the study. These thresholds were introduced to eliminate the influence of the learning curve during the experiment.

Participants are blinded toward the aim of the study. They are instructed to test the familiar curriculum in an IVR environment but are unaware of possible distractions. Prior research has shown simulation to be a useful tool not only for training practical skills, but also regarding communication, stress, and handling of difficult situations [15].

The results may suggest how to improve the work environment in the OR by identifying distractions, and classifying and reducing them, wherever possible. As mentioned earlier, however, complete elimination is nearly impossible, but reduction should be attempted especially during critical steps of the operation. Different surgeons react differently to stress and distractions [16] and experienced surgeons appear to be less impaired by them [7]. This suggests that the individual reaction to stress may be a subject to training, which could be conducted in VR.

### Objectives

The primary objective of this study is to examine the influence of simulated distractions in IVR on performance in laparoscopic surgery. The LapSim with haptic feedback is able to evaluate laparoscopic performance using the time required for an exercise, tissue handling, amount of bimanual work, and instrument path length. Secondary objectives are to collect participant-specific data, such as grade of immersion, subjective perception of the passing of time, subjective perception of stress in the form of the National Aeronautics and Space Administration Task Load Index (NASA TLX) questionnaire, system usability scale, visually induced motion sickness (VIMS), eye strain, heart rate, and eye tracking behavior.

### Trial Design

The trial is designed as a cross-over trial. Each participant trains in 6 exercises on the simulator until a proficiency cutoff level is reached. After the training phase, participants enroll in the experiment. They complete the exercises wearing an HMD in IVR in a 360° video of the OR while 5 different auditory, visual, and audio-visual distractions are played. Each exercise is completed once combined with all 5 distractions and twice without distractions, amounting to 42 exercises in total. The order of exercises and distractions is determined by a Latin squares design, which minimizes potential order effects as each participant has an individual order of exercises and distractions [17]. The sequences are prepared with control for neighboring exercises and distractions, ensuring that there is no clustering of the same exercises or distractions.

### Study Setting

The study will be conducted as a single-center study in the University Medical Center Mainz, Germany. The Department of General Visceral and Transplant Surgery is a high-volume center for minimally invasive surgery and has implemented mandatory laparoscopy training for all residents. Facilities for training are available. The department conducts mandatory laparoscopy training for all medical students and offers an elective about minimally invasive surgery.

### Eligibility

#### Overview

Eligible are volunteer medical students of the University Medical Center Mainz, who have completed the training of the 6 laparoscopic exercises to a predetermined proficiency level as well as residents in visceral surgery and gynecology after completion of the department-specific laparoscopy training.

#### Inclusion and Exclusion Criteria

Since the presented program is a novelty design there is no effect size known. Due to prior experience, a sample size of 30 medical students and 20 residents in visceral surgery and gynecology was chosen. Medical students are recruited through announcements via posters and flyers in the University Medical Center Mainz, as well as during classes in visceral surgery. Surgical and gynecological residents are informed about the study on a personal basis and recruited if interested. To improve adherence to the experiment, breaks are offered after each exercise and can be taken at the liberty of the participants. The experiment can be paused or discontinued at any time if the participant wishes to, the number and duration of additional pauses will be recorded.

Exclusion criteria are impacted sight or hearing, which are tested before inclusion using standardized tests. All participants must be able to stand for 120 minutes upright to work on the laparoscopy simulator and are excluded if this is not possible. Furthermore, all participants have to complete a laparoscopy curriculum and reach a threshold to rule out the learning curve as an effect on the study. Not passing the threshold is an exclusion criterion.

### Data Collection and Management

Data are collected immediately after the experiment using LimeSurvey (LimeSurvey GmbH, licensed by the Johannes Gutenberg-University Mainz) and later transferred to SPSS (version 27; IBM Corp).

Data are checked manually for correct transfer. Data are going to be stored in anonymized fashion on password-protected computers of the study group in locked rooms, to which only members of the study group have access to.

### Ethical Considerations

The trial including all information, and the consent form has been reviewed and approved by the local ethics committee (Ethik-Kommission der Landesärztekammer Rheinland-Pfalz, application: 2022-16528-andere Forschung erstvotierend). Each eligible participant will be informed concisely about the study's potential and risks. If participation is desired, written consent is obtained by a member of the study group before inclusion in the trial. All participants have the option to withdraw their consent or quit the study at any time. All data is handled confidentially and in an anonymized fashion. There is no compensation for participants.

### Interventions

Performance in laparoscopic exercises will be evaluated with and without different distractions. A cross-over design was chosen to allow within-subject control for each participant. Primary comparators are performance in laparoscopic surgery during different distractions. This will be assessed by the parameters of the laparoscopic simulator. Prior studies have shown more experienced surgeons to be less impacted by distractions [7], deeming the 2 eligible groups—medical students and surgical residents—an interesting contrast. Further comparators are subjective passing of time, subjective stress levels, and heart rate [18-20], as well as usability, VIMS, eye strain, and immersion, which are key comparators in newly developed VR programming [21-23]. Furthermore, the Self-Assessment Manikin score is assessed to employ a measurement for psychological stress and emotion [24]. Most of the aforementioned parameters are assessed by validated questionnaires, which have been used in similar experiments for years. Eye tracking is performed using HMD, to examine whether participants look at distractions without consciously perceiving them.

**Figure 2:**
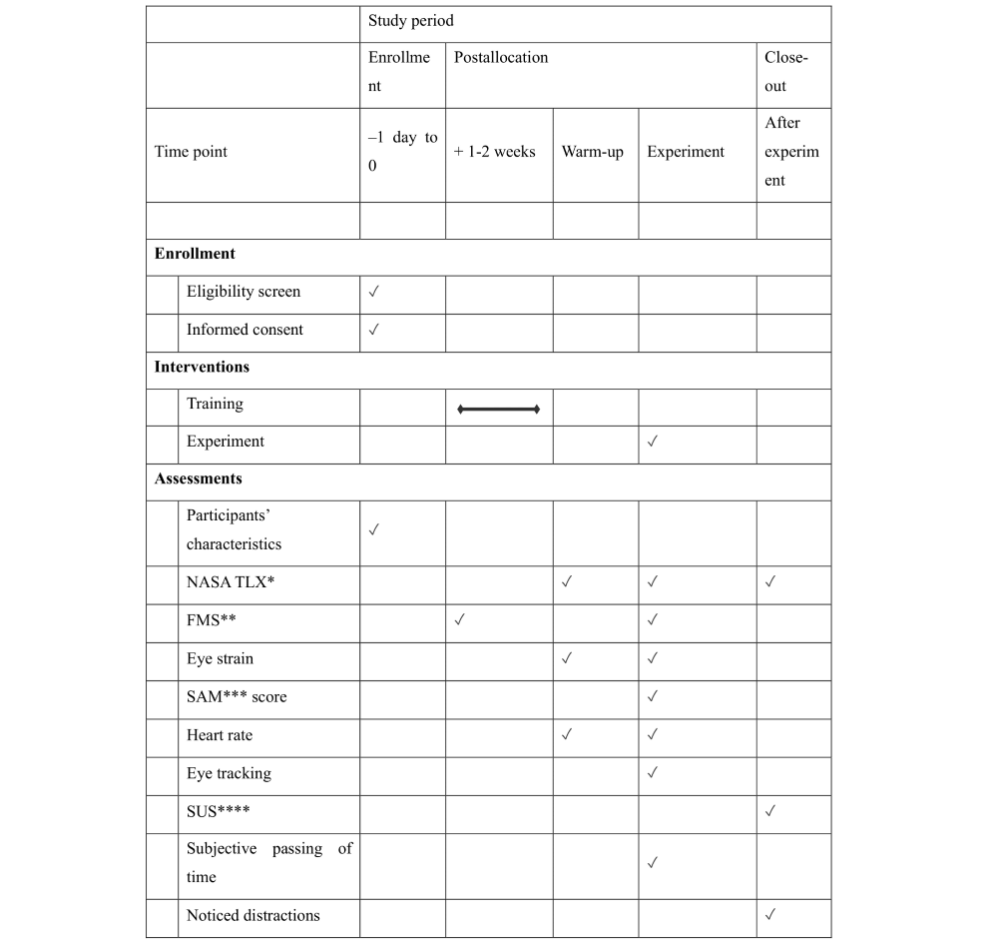
The course of the study with its different phases and the data collected at different time points. Before enrolling in the study, participants complete a mandatory laparoscopy curriculum. Right before the experiment, a warm-up phase is conducted. During the experiment, data about performance on the laparoscopy simulator as well as heart rate and the items of multiple questionnaires are collected. After completion of the experiment participants evaluate the distractions as well as usability of the system. *NASA TLX: National Aeronautics and Space Administration Task Load Index. **FMS: Fast Motion Sickness Scale. ***SAM: Self-Assessment Manikin. ****SUS: system usability scale.

Each participant is going to complete each of the 6 laparoscopic exercises with all 5 distractions and twice without distraction. This amounts to 42 exercises, breaks will be taken after exercises 14 and 28. Mandatory breaks are 10 minutes long but can be extended as needed. Additional breaks will be offered after each exercise and can be taken at the freedom of the participant. The order of exercises and distractions is determined by a Latin squares design. After each exercise, participants are asked for their subjective stress levels using the NASA TLX score, subjective passage of time, VIMS (measured by the Fast Motion Sickness Scale [17]), and eye strain. After the experiment, participants are questioned about usability and immersion and are asked if they have noticed any distractions and, if yes, of which kind and how disruptive they were perceived to be. The trial design is depicted schematically in Figure 2 with all data collected at different time points.

### Statistical Methods

Statistical analysis of the primary outcome, perceived passage of time, and perceived stress will be conducted using the Wilcoxon test since it consists of a connected sample. Participant-related data, usability, immersion, and visually induced motion sickness are analyzed using repeated-measures ANOVA and 1-tailed *t* tests. Eye tracking is analyzed qualitatively (ie, the participant looked away from the laparoscopy monitor: yes or no). The students’ and residents’ performance, subjective passing of time, and NASA TLX variance over different time points and exercises will be analyzed using ANOVA.

## Results

The trial started recruitment on August 31, 2022 and is still ongoing. By July 2024, the trial had recruited 30 medical students and 9 surgeons and is still recruiting. Recruitment of surgeons has been progressing slowly because of high demand and workload in the department, thus inclusion has been prolonged.

## Discussion

This study aims to explore the effect of different distractions during laparoscopic surgery using laparoscopic simulation in high IVR. The OR can be a demanding and at times stressful workplace. Distractions during surgery are often triggered by necessary communication and urgent incoming demands [4], which cannot be eliminated even with utmost consideration and thoughtfulness. Furthermore, teaching, organizational tasks, and care for emergencies and complications disrupt the workflow, without the option for delay.

This trial is the first evaluation of distractions in the OR using highly immersive VR and may be able to assist in the possible improvement of working conditions and environments as well as the development of specific training. A group of 50 participants is sufficient for the exploratory aim of the study and according to experience attainable. Novel technologies like VR using HMDs usually spark the interest of possible participants and others alike but have not found extensive everyday application, yet. Simulation has been known to be a useful resource for years not only in laparoscopy [1,15]. If a laparoscopic curriculum extended by highly VR can be tested successfully, implementation as a set curriculum for the improvement of more than only manual skills could be possible.

Limitations of this trial include the usability of VR HMDs, which can be distracting and exhausting to wear to some participants while performing complex tasks. This might reduce immersion during training. However, we have not experienced any difficulties with this yet.

Prior research suggests, that more experienced surgeons are able to handle demanding situations more easily and are able to multitask [7]. This knowledge offers an opportunity for targeted training of stress resistance and multitasking. The future use of this program could include standardized training of medical students and surgical residents. Furthermore, a future extension of the program could facilitate an even more high-stress environment simulating an unstable patient and including different distractions that are common in an emergency situation. This would make the program interesting for more advanced surgeons as well.

## Abbreviations

HMD: head-mounted display
IVR: immersive virtual reality
NASA TLX: National Aeronautics and Space Administration Task Load Index
OR: operating room
VR: virtual reality
VIMS: visually induced motion sickness

## Data Availability

The data sets used and/or analyzed during the current study are available from the corresponding author on reasonable request in a condensed and thus not trackable manner. The data acquired during the trial will be kept strictly confidential and only be accessible to members of the research group. The personal information of participants will not be released, and their privacy will be protected.
